# Supplementary material for: RE-PERG in early-onset Alzheimer’s disease: A double-blind, electrophysiological pilot study
Source: PLoS One. 2020 Aug 13;15(8):e0236568. doi: 10.1371/journal.pone.0236568 (PMC7425894; doi:10.1371/journal.pone.0236568)
Supplement: Attachment — Submitted filename: Response to Reviewers.docx [file pone.0236568.s001.docx]

Dear Editor,

Enclosed you find the updated version of our manuscript, which has been widely revised according to your reviewers' criticisms. The manuscript has also been corrected by an English medical editor.

Best regards

5. Review Comments to the Author

Please use the space provided to explain your answers to the questions above. You may also include additional comments for the author, including concerns about dual publication, research ethics, or publication ethics. (Please upload your review as an attachment if it exceeds 20,000 characters)

Reviewer #1: Introduction:
1. Authors should explain in more detail what Alzheimer's disease is and how it differs from (VD) -related MCI, describe changes in the CNS, retina, and why eye biomarkers may be useful.

 DONE: please, see new introduction

2. How do we confirm the diagnosis of AD in sMRI?

DONE: please, see introduction, lines 93-95

MATERIALS AND METHODS:
1. The authors described that neurologic exclusion criteria were: „neurological / psychiatric conditions other than mild AD"- what about patients who had vascular dementia? After all, they were included in the study.

DONE: line 149

2. Did all participants have an MRI? How on the basis of sMRI diagnosed and distinguish patients with AD and VD.

DONE: please, see introduction and lines 165-166

3. The description of the statistics should be more detailed, paying attention to the tests used.

DONE: lines 219-228

Results:
1. „ANOVA analysis showed no difference between groups for age. MD. PSD RNFL and GCC.”- errors in punctuation

DONE: lines 249-251

2. Please specify the number and characteristics of excluded subjects to reduce the selection bias.

DONE: lines 239-241

Discussion:
1. Epidemiology, diagnosis and pathogenesis should be included in the introduction (not in the discussion).

DONE: please, see new introduction

2. Unfortunately, the most effective tests for the diagnosis of AD are expensive and invasive.”- e.g. MRI is not an invasive test, therefore this sentence should be formulated more precisely

DONE: please, see lines 269-270

3. "Since the neurodegeneration of RGCs shows similar features in both glaucoma and AD, we performed this study in order to evaluate the ability of RE-PERG in the identification of the early stage of AD. "- How can RE-PERG distinguish AD from glaucoma? Since the PERG test is used in glaucoma diagnosis why the authors did not compare glaucoma, AD and HC?

This is not the aim of our study. This matter is developed in the discussion section; currently no diagnostic test used for glaucoma (i.e. OCT of the optic nerve, visual field examination, electrophysiological tests) can distinguish between glaucoma and AD, this is true also for REPERG. According to some works the only difference seems to be the appareance of the optic nerve (which should show cupping in glaucoma but not in AD) even if according to other authors a similar cupping can be observed also in AD. As we state in the discussion section, this situation can make it difficult to distinguish between glaucoma and AD, especially when a raise of intraocular pressure is not detectable, that is, in case of normal-tension glaucoma suspect.

4. The authors did not describe the limitations of their research

DONE: please, see lines 317-325

5. MMSE may not be the best cognitive test with which to measure AD related cognitive impairment. This should be acknowledged and the limitations explored in the discussion.

DONE: please, see lines 317-325

6. Why do the authors compare patients with VD to AD and NC if the problem is to distinguish AD from glaucoma? I think that vascular changes in the retina in AD patients should be mentioned (Bulut, M., Kurtuluş, F., Gözkaya, O., Erol, M. K., Cengiz, A., Akıdan, M., & Yaman, A. (2018). Evaluation of optical coherence tomography angiographic findings in Alzheimer’s type dementia. British Journal of Ophthalmology, 102(2), 233-237.) and describe differences in microvascularization between AD, POAG and NC (Zabel, P., Kaluzny, J. J., Wilkosc-Debczynska, M., Gebska-Toloczko, M., Suwala, K., Zabel, K., ... & Araszkiewicz, A. (2019). Comparison of Retinal Microvasculature in Patients With Alzheimer's Disease and Primary Open-Angle Glaucoma by Optical Coherence Tomography Angiography. Investigative ophthalmology & visual science, 60(10), 3447-3455.)

DONE: please see our answer to point 3. As for OCT-A, see lines 342-351

References:
1. Kamila K., Wojciech L., Andrzej P. Pattern electroretinogram (PERG) and pattern visual evoked potential (PVEP) in the early stages of Alzheimer’s disease Doc Ophthalmol. 2010 435 Oct; 121(2): 111–121.”- these are not the author's' last names

DONE

Reviewer #2: Manuscript n.: PONE-D-19-31892

TITLE: RE-PERG in early-onset Alzheimer’s disease. A double-blind electrophysiological pilot study.

Article Type: Research Article.

In this study, the Authors investigate the ability RE-PERG (retest PERG) in detection inner retinal bioelectric function abnormalities in patients with early-onset Alzheimer Disease (AD). They investigated a series of pt. (17 pt.) with AD and report mean RE-PERG amplitude significantly lower and phase of 2nd harmonic (PERG SDPh) higher in AD pts. compared with controls. Moreover, they affirm that RE-PERG can be useful to identify early AD stages and that changes may be imputable to magnocellular pathway dysfunction not present in other conditions, like vascular dementia and conclude that RE-PERG could be a new promising biomarker of neurodegenerative disease.

The study is well conducted and written, results are clear, sound well and could be interesting for the readership of the journal, even if not at all new (see previous paper on the same field reported in references).
The manuscript has some issues needed to be addressed.

Major criticism are:
- First, the AA address the magnocellular pathway (M pathway), but it should be kept in mind that there are other pathway subsystems (Parvo-, P and Konio-cellular, K subsystem; see Paper of Livingstone, Porciatti, van Essen etc. on this topic). The AA should add some information concerning these subsystems and their propriety. In fact whereas color information is processed mainly by the P system, luminance by both P and M subsystem. Moreover, the stimulus employed by the AA is not selective for the Magno and result could be aspecific. The AA should add some informations on visual pathways subsystem and property.

DONE: please, see new introduction (lines 71-83)

- Second the AA to exclude a bias in their study should exclude involvement of other subsystem before to affirm an exclusive magnocellular dysfunction in their conclusions. Please clarify.

Please please, see new introduction and comments (lines 77-80)

- Third, How can the AA exclude that a lower RE-PERG and a higher SDPh RE-PERG are not an aspecific changes

By means of exclusion criteria

Keywords: I suggest add visual pathway subsystem.

DONE

Abstract: modify according the above criticisms.
DONE: please, see lines 317-325

Introduction: see major criticisms.
In line 89 they affirm that “ … this parameter is not influenced by optical …”; I suggest “scarcely influenced by refraction “

Our statement is based on exclusion criteria and on our previous works (ref 56-58)

Materials and methods: -

Discussion and Concluding remarks: they have to be rewritten following the above suggestions and comments.

DONE

References: there are only few typing mistaken and two reference in my opinion seems the same (n. 14 and 56): check.

DONE
