# Supplementary material for: RE-PERG in early-onset Alzheimer’s disease: A double-blind, electrophysiological pilot study
Source: PLoS One. 2020 Aug 13;15(8):e0236568. doi: 10.1371/journal.pone.0236568 (PMC7425894; doi:10.1371/journal.pone.0236568)
Supplement: Attachment — Submitted filename: Response to reviewers.docx [file pone.0236568.s002.docx]

6. Review Comments to the Author

Please use the space provided to explain your answers to the questions above. You may also include additional comments for the author, including concerns about dual publication, research ethics, or publication ethics. (Please upload your review as an attachment if it exceeds 20,000 characters)

Reviewer #1: All the concerns have been addressed. The authors corrected the manuscript in accordance with the suggestions of the reviewers, which made the manuscript suitable for publication.

Reviewer #2: Manuscript n.: PONE-D-19-31892

TITLE: RE-PERG in early-onset Alzheimer’s disease. A double-blind electrophysiological pilot study.

Article Type: Research Article.

I revised again with pleasure the interesting manuscript that in many aspects has been significantly improved. The Authors were able to overcome most of criticisms.
Therefore I only have only two minor comments:

-Changes in Re-PERG were they also accompanied in parallel by changes in the so-called apparent latency or not?

The apparent latency is evaluated by measuring the phase as a function of temporal frequency, i.e. using different temporal frequencies; in this study we used only one temporal frequency (please, see methods), therefore the apparent latency was not evaluated

-The main conclusion that Re-PERG changes may be attributable predominantly to magnocellular pathway should be softened because the other two visual stream (Konio and Parvo) were not investigated (clarified: see lines 43, 288, 299-301); in my opinion their study prove an alteration in RE-PERGs more evident in AD compared with VD. Otherwise it seems that the AA want to prove forcedly that an involvement of the RE-PERG means diagnosis of AD and instead may also be present in other diseases or depend from common visual changes age-related, e.g. presbyopia, cataract and so on. We had widely discussed problems related to the differential diagnosis between glaucoma and AD based on several diagnostic tools, including REPERG. As for the other conditions, see lines 350-355

**Comments to the Author**

1. If the authors have adequately addressed your comments raised in a previous round of review and you feel that this manuscript is now acceptable for publication, you may indicate that here to bypass the “Comments to the Author” section, enter your conflict of interest statement in the “Confidential to Editor” section, and submit your "Accept" recommendation.

Reviewer #1: All comments have been addressed

Reviewer #2: (No Response)

2. Is the manuscript technically sound, and do the data support the conclusions?

The manuscript must describe a technically sound piece of scientific research with data that supports the conclusions. Experiments must have been conducted rigorously, with appropriate controls, replication, and sample sizes. The conclusions must be drawn appropriately based on the data presented. 

Reviewer #1: Yes

Reviewer #2: Yes

3. Has the statistical analysis been performed appropriately and rigorously? 

Reviewer #1: Yes

Reviewer #2: Yes

4. Have the authors made all data underlying the findings in their manuscript fully available?

The [PLOS Data policy](http://www.plosone.org/static/policies.action#sharing) requires authors to make all data underlying the findings described in their manuscript fully available without restriction, with rare exception (please refer to the Data Availability Statement in the manuscript PDF file). The data should be provided as part of the manuscript or its supporting information, or deposited to a public repository. For example, in addition to summary statistics, the data points behind means, medians and variance measures should be available. If there are restrictions on publicly sharing data—e.g. participant privacy or use of data from a third party—those must be specified.

Reviewer #1: Yes

Reviewer #2: Yes

5. Is the manuscript presented in an intelligible fashion and written in standard English?

PLOS ONE does not copyedit accepted manuscripts, so the language in submitted articles must be clear, correct, and unambiguous. Any typographical or grammatical errors should be corrected at revision, so please note any specific errors here.

Reviewer #1: Yes

Reviewer #2: Yes

6. Review Comments to the Author

Please use the space provided to explain your answers to the questions above. You may also include additional comments for the author, including concerns about dual publication, research ethics, or publication ethics. (Please upload your review as an attachment if it exceeds 20,000 characters)

Reviewer #1: All the concerns have been addressed. The authors corrected the manuscript in accordance with the suggestions of the reviewers, which made the manuscript suitable for publication.

Reviewer #2: Manuscript n.: PONE-D-19-31892

TITLE: RE-PERG in early-onset Alzheimer’s disease. A double-blind electrophysiological pilot study.

Article Type: Research Article.

I revised again with pleasure the interesting manuscript that in many aspects has been significantly improved. The Authors were able to overcome most of criticisms.
Therefore I only have only two minor comments:

-Changes in Re-PERG were they also accompanied in parallel by changes in the so-called apparent latency or not?

-The main conclusion that Re-PERG changes may be attributable predominantly to magnocellular pathway should be softened because the other two visual stream (Konio and Parvo) were not investigated; in my opinion their study prove an alteration in RE-PERGs more evident in AD compared with VD. Otherwise it seems that the AA want to prove forcedly that an involvement of the RE-PERG means diagnosis of AD and instead may also be present in other diseases or depend from common visual changes age-related, e.g. presbyopia, cataract and so on.
